# Role of miRNA in Highly Pathogenic H5 Avian Influenza Virus Infection: An Emphasis on Cellular and Chicken Models

**DOI:** 10.3390/v16071102

**Published:** 2024-07-09

**Authors:** Dibakar Chowdhury, Md. Nayeem, Hillary A. Vanderven, Subir Sarker

**Affiliations:** 1 Laboratory of Influenza Research, College of Veterinary Medicine, Chungnam National University, Daejeon 34134, Republic of Korea; chowdhurydibakar565@gmail.com; 2 One Health Institute, Chattogram Veterinary and Animal Sciences University, Khulshi, Chattogram 4225, Bangladesh; mdnayeem4567@gmail.com; 3 Biomedical Sciences & Molecular Biology, College of Public Health, Medical and Veterinary Sciences, James Cook University, Townsville, QLD 4811, Australia; hillary.vanderven@jcu.edu.au; 4 Australian Institute of Tropical Health and Medicine, James Cook University, Townsville, QLD 4811, Australia; 5 Department of Microbiology and Immunology, Peter Doherty Institute for Infection and Immunity, University of Melbourne, Parkville, VIC 3010, Australia

**Keywords:** avian influenza virus, H5 subtypes, miRNA

## Abstract

The avian influenza virus, particularly the H5N1 strain, poses a significant and ongoing threat to both human and animal health. Recent outbreaks have affected domestic and wild birds on a massive scale, raising concerns about the virus’ spread to mammals. This review focuses on the critical role of microRNAs (miRNAs) in modulating pro-inflammatory signaling pathways during the pathogenesis of influenza A virus (IAV), with an emphasis on highly pathogenic avian influenza (HPAI) H5 viral infections. Current research indicates that miRNAs play a significant role in HPAI H5 infections, influencing various aspects of the disease process. This review aims to synthesize recent findings on the impact of different miRNAs on immune function, viral cytopathogenicity, and respiratory viral replication. Understanding these mechanisms is essential for developing new therapeutic strategies to combat avian influenza and mitigate its effects on both human and animal populations.

## 1. Introduction

Influenza viruses are members of the Orthomyxoviridae family, comprising four genera depending on antigenic variations among nucleocapsid (NP) and matrix (M) proteins: *Alphainfluenzavirus* (influenza A virus), *Betainfluenzavirus* (influenza B virus), *Gammainfluenzavirus* (influenza C virus), and *Deltainfluenzavirus* (influenza D virus) [1,2,3]. Influenza A viruses (IAVs) possess two surface glycoproteins, namely hemagglutinin (HA) and neuraminidase (NA), which play critical roles in virus infectivity and replication. Influenza A viruses are further categorized into subtypes based on the antigenicity of HA and NA, with 18 known HA subtypes (H1–H18) and 11 known NA subtypes (N1–N11). Influenza virus HA binds to sialic acid on host cells and leads to the fusion of the enveloped virus with the cell membrane, which is a vital stage in the viral life cycle [4]. IAVs are also capable of causing serious illness and seasonal outbreaks in humans, with the capacity to spark a global pandemic [5]. All viruses that cause avian influenza (AI) are IAVs [6]. Avian influenza virus, particularly H5N1, outbreaks are affecting both domestic and wild birds on an enormous scale, and it has already spread to mammals on multiple occasions (e.g., mink, dairy cattle, and humans) [7,8,9]. Around 193.9 million birds have died as a result of the H5N1 and H5N8 outbreaks since 2020. The majority of these deaths were in wild birds migrating from Europe to the Middle East that came into contact with the virus from domestic poultry [10]. In this review, the role of microRNAs in highly pathogenic avian influenza (HPAI) virus infection is described.

MicroRNAs (miRNAs), typically around 20 to 25 nucleotides in size, are small RNA molecules naturally occurring within cells of a variety of organisms, including both animals [11,12,13,14] and plants [15,16,17,18]. MiRNA sequences’ biogenesis has been largely explained [19]. A distinctive stem-loop sequence known as a pre-mir, which may then be removed from a longer primary transcript known as a pri-mir, is processed to produce the mature miRNA (referred to as miR). Evidence indicates that miRNAs are transcribed by RNA polymerase II, and the resulting transcripts are capped and polyadenylated, yet only a small number of primary transcripts have been thoroughly characterized [20]. MiRNAs are identified using a combination of previously published criteria for both their expression and biogenesis in order to avoid classifying siRNAs or pieces of other RNAs as miRNAs [21]. The “miR” prefix and a distinct identification number (such as miR-1, miR-2, and miR-89, etc.) are used to name microRNAs. The same three-letter prefix, capitalization, hyphenation, and italics are used in the naming of the genes that encode the miRNA in accordance with the organism’s conventions [21]. Among the databases dedicated to miRNAs, the miRBase database is the first automated pipeline for identifying miRNA target genes in different animal genomes, combining the functions of a sequence database and gene naming that were previously performed by the microRNA Registry [20,22].

MiRNAs have significant roles in both the normal and abnormal functions of airway cells, including pulmonary growth, immune response, fibrosis, and cancer development [23]. MiRNAs are post-transcriptional regulators of gene expression. They target specific RNA sequences and control protein production by binding to the untranslated region of messenger RNA (mRNA) [15]. MiRNAs regulate various host–IAV interactions, such as immune responses, cell cycle control, and apoptosis. Interactions between host miRNAs and IAV also control virus replication, translation, and pathogenicity. For example, IAV infection decreases the levels of miR-4276 and miR-200a, leading to the apoptosis of infected cells and a compromised inflammatory response [24], respectively. Numerous miRNAs, like miR-650, play roles in antiviral defense by targeting either host or viral genes [25]. Furthermore, certain miRNAs are crucial in modulating pro-inflammatory signaling pathways during IAV infection. For instance, miR-29c and miR-451 levels rise following IAV infection to suppress the inflammatory response of cells [26,27]. Viral infection either triggers or inhibits the expression of cellular miRNAs, which in turn modulate the host response to infections. Two human miRNAs, namely miR-136 and miR-507 have demonstrated a potential binding affinity for IAV genes responsible for encoding the polymerase basic 2 (PB2) and hemagglutinin (HA) proteins [28]. Further, miR-323, miR-491, and miR-654 suppress the replication of an H1N1 IAV by controlling the BAX inhibitor gene [14]. Following influenza virus infection, some miRNAs are crucial in preparing airway cells for repair and regeneration [29]. Many cellular miRNAs are differentially expressed during the IAV infection of host cells. As a result, the up- or downregulation of specific miRNAs during IAV infection may serve as indicators for the pathophysiology or severity of the influenza illness. Certain miRNAs are involved in modulating translation (miR-9 increases IAV M1 and NP protein expression) [30], while others control transcription (miR-485 suppresses the PB1 transcript by directly binding to the viral genome, which leads to the inhibition of IAV replication) [31]. The infection pattern of an IAV could be determined by examining the expression levels of cellular miRNAs; yet, certain miRNAs target a single IAV subtype, while others target multiple IAV subtypes.

Taken together, the findings suggest that influenza virus infection can induce or suppress the production of miRNAs in various cells, including lung epithelial cells, alveolar macrophages, and peripheral blood mononuclear cells (PBMCs), etc., which, in turn, either promote or inhibit viral pathogenesis and replication. Therefore, the most promising candidates for the development of miRNA-based treatments for influenza illness may be cellular miRNAs that target genes involved in immune response, apoptosis, and proteases such as furin that facilitate IAV entry into the cell. However, due to the immunopathogenic nature of influenza virus infection, caution must be exercised. In this review, we describe current research indicating that miRNAs play a significant role in HPAI virus (HPAIV) infections caused by H5 subtype viruses. We discuss the potential impact of these miRNAs on the immune system, virus cytopathogenicity, and viral replication. Understanding the function of miRNAs in HPAIV infection could enhance our understanding of the mechanisms of IAV infection and suggest possible avenues for future miRNA-based therapies.

## 2. In Vitro Study of H5 Subtype Highly Pathogenic Avian Influenza Virus (HPAIV)

### 2.1. miRNA Studies in A549 Human Lung Epithelial Cells

**Figure 1:**
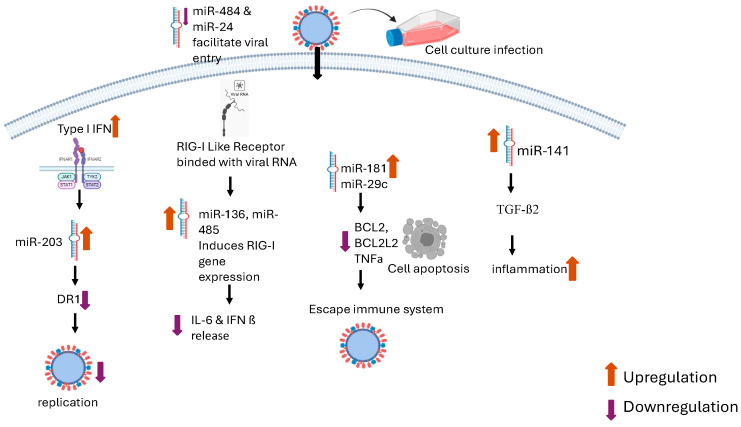
Overview of involvement of microRNAs in different host-mediated pathways against HPAIV infection. The figure shows how microRNAs influence the virus’s entry and affect various immune response signaling pathways, leading to the differential expression of several cytokines and apoptotic factors.

A549 cells were infected with either HPAIV H5N1 or the pandemic 2009 H1N1 influenza virus to examine fluctuations in the levels of cellular miR-203. Both H5N1 and H1N1 IAVs triggered an increase in expression of miR-203 in A549 cells. They also investigated the contribution of type I interferon (IFN) IFN to increased expression of miR-203 during IAV infection by exposing A549 cells to IFN-α alone and subsequently extracting total RNA to assess the levels of miR-203. There was an induction of miR-203 expression by IAV infection, but type I IFN also strongly stimulated the expression of miR-203. Luciferase assay results confirmed that the expression of miR-203 in IAV-infected A549 cells was strongly induced at the transcriptional level by type I IFN [32]. Two transcription factors linked to the IFN signaling pathway were the predicted cellular targets of miR-203: Interferon-stimulated gene factor 3 (ISGF3) and Nuclear Factor kappa B (NF-κB). The ISGF3 complex plays a crucial role in the IFN-stimulated JAK-STAT signaling pathway, consisting of a signal transducer and activator of transcription 1 (STAT1), STAT2, and IRF9 [33]. Furthermore, the NF-κB pathway is stimulated by signaling cascades activated by IFN [34,35]. These two transcription factors, ISGF3 and NF-κB, may play a significant role in triggering miR-203 expression and require further investigation (Figure 1). Additionally, their study findings showed a correlation between DNA demethylation of the promoter region and miR-293 upregulation upon H5N1 infection in A549 and Vero cells. DNA Methyl Transferase 1 (DNMT1) has a significant role in DNA demethylation during H5N1 infection, and this demethylation process could induce the expression of miR-203 regardless of the presence of type I IFNs. Moreover, their results revealed that introducing miR-203 externally suppresses the replication of H5N1 HPAIV in A549 cells. The production of viral genomic RNA during the initial phase of virus infection also appears to be more effective in cells lacking miR-203 [32]. This study reports host gene DR1 (downregulator of transcription 1) as a new target for miR-203. DR1 supports the replication of IAV by inhibiting IFN production and directly promoting viral RNA replication through interaction with the viral RNA-dependent RNA polymerase (RdRp) [36]. Zhang et al. demonstrated that silencing DR1 using small interfering RNA (siRNA) reduced the level of endogenous DR1 expression and hindered the replication of IAV, confirming that miR-203 restricts IAV replication by targeting DR1 (Table 1). The outcome of this investigation underscores the potential of miRNAs as a therapeutic approach for treating IAV infection [32].

RIG-I is a cytosolic pattern recognition receptor (PRR) that detects viral RNA and triggers antiviral signaling [37,38,39]. Another study focusing on the interaction between miRNA and RIG-I in A549 cells suggests that miRNA-136 plays a dual role in post-transcriptional regulation and immune activation by modulating innate immunity against avian influenza virus infection. They propose that the induction of miR-136 may have a significant impact on H5N1 virus infection through interactions with the cytosolic pattern recognition receptor RIG-I. MiR-136 was identified as an endogenous activator of RIG-I, potentially aiding in the control of viral infections [40]. MicroRNAs also have the capability of triggering pro-inflammatory cytokine production [41]. In A549 cells, miR-136 increased the secretion of IL-6 and IFN-β compared with other miRNAs, like miR21, miR29a, and let7 (Figure 1) [42,43]. While all of these miRNAs have immunostimulatory properties, the precise structural characteristics of miR-136 that promote the activation of RIG-I and the underlying mechanistic differences remain an area of significant interest. MiR-136 is described as a suppressor of IAV replication in vitro, suggesting its potential as a promising treatment for IAV and other viral infections (Table 1) [40]. MiR-188-3p has also been reported to inhibit the replication of IAVs that can infect humans, such as the H5N6 and H7N9 subtypes, by targeting the mRNA of the PB2 gene. MiR-188-3p reduces PB2 expression by binding to predicted sites in the PB2 mRNA transcript. This effectively inhibits the replication of IAVs (including H1N1, H5N6, and H7N9) in A549 cells. These findings indicate that cellular miR-188-3p could be employed for RNAi-based therapeutic strategies against IAV (Table 1) [44]. MiR-21-3p was found to be downregulated during H5N1 IAV infection and may enhance influenza virus replication in A549 cells [45]. Furthermore, the H5N1 IAV’s PB2 gene contains putative miRNA binding sites that may suppress H5N1 viral replication. The binding sites for miR-584-5p and miR-1249 are located inside the PB2 gene of H5N1. Both of these miRNAs downregulate PB2 expression in A549 cells, which prevents H1N1 and H5N1 IAV from replicating (Table 1). However, the HPAI H5N1 virus may be able to counteract this suppressive effect by downregulating miRNAs that target its viral RNA [46].

A human microRNA profiling study using A549 cells reported that 15 common miRNAs, including the let-7 family, miR-10, miR-15, miR-21, miR-29, miR-30, miR-101, miR-132, miR-148, and miR-548, were differentially expressed during H5N1 HPAIV infection. The H5N1 HPAIV infection led to the greatest changes in miRNA expression compared with uninfected A549 cells (Table 1). From this group of differentially expressed miRNAs, some miRNAs (miR-let-7, miR-29, and miR-128) are only capable of targeting a single gene of H5N1 IAV. However, miR-29 can also target cellular BCL2L2, an apoptosis-related gene. Other target predictions showed that miR-660 and miR-128 target the PA gene of H5N1 IAV, while miR-29a targets the HA gene. On the other hand, some miRNAs, such as miR-548 and miR-16, can target multiple influenza virus genes [47]. MiRNAs involving the let-7 family, miR-30a-c, miR-132, and miR-30e can target various genes involved in lung repair (Table 1) [27,48]. The upregulation of miR-181c can suppress numerous genes related to cellular immune defense, such as BCL2, IL-2, and TNFα. The downregulation of these host genes enables the influenza virus to evade the suppressed host immune system (Table 1 and Figure 1) [49,50,51]. Another miRNA of interest is miR-29c, which can induce cell apoptosis and is upregulated during H5N1 infection. Substantial evidence indicates that this miRNA plays a role in virus-induced apoptosis by repressing the anti-apoptotic agent BCL2L2 (Table 1) [52]. Interestingly, miR-484 is also upregulated in cells infected with H5N1 and H3N2 IAVs. This miRNA inhibits furin, a proteolytic enzyme that cleaves the HA0 protein and facilitates influenza virus entry into host cells [53,54].

MiR-24, which is part of the miR-23 cluster, is a highly conserved microRNA found in multiple vertebrates and is widely expressed across multiple tissues [55,56]. The role of miR-24 as a post-transcriptional regulator was investigated using A549 cells. Specifically, efforts were made to understand how miR-24 affects the cleavage of HA0 by furin. The downregulation of furin-directed miRNAs by HPAIVs may increase furin expression, thereby enhancing furin-mediated proteolytic cleavage of HA0 glycoproteins and increasing the generation of infectious virions. This study concentrated on the role of miR-24 during H5N1 IAV infection, given the pandemic potential of this virus and the presence of the optimal furin cleavage sequence R-X-K/R-R between the HA1 and HA2 subunits [57,58,59]. The rapid, temporary induction of miR-24 after H5N1 IAV adsorption may be stimulated by activation of the NF-κB signaling pathway, leading to the expression of miR-23b–27b–24 cluster members in A549 cells [60,61,62]. The overexpression of miR-24 decreases both furin mRNA levels and its enzymatic activity in human A549 cells. Administration of exogenous miR-24 also diminishes the release and spread of H5N1 HPAIV in A549 cells [60].

**Table 1:** Differential expressions of microRNAs against HPAIV and their target sites revealed in various studies using cell models.

microRNA	Expression	H5 Subtypes	Cells	Target	Ref.
miR-203	Upregulation	A/Vietnam/1194/2004 (H5N1)	A549	DR1	[32]
miR-136	Upregulation	A/chicken/Hubei/327/2004 (H5N6)	A549	IL6	[40]
miR-188-3P		A/chicken/Hubei/XY918/2016(H5N6)	A549	PB2	[44]
miR-345				PB2	[44]
miR-3183				PB1	[44]
miR-769-3p				NP	[44]
miR-15a-3p				PA	[44]
miR-21-3p	Downregulation	A/goose/Jilin/hb/2003 (H5N1)	A549	HDAC-8	[45]
let 7	Upregulation	A/Thailand/NK165/2005 (H5N1)	A549	Unknown	[47]
miR-10	Downregulation			Unknown	[47]
miR-15	Upregulation			Unknown	[47]
miR-21	Upregulation			Unknown	[47]
miR-29c	Upregulation			BCL2L2	[47]
miR-29a	Upregulation			HA	[47]
miR-30	Upregulation			Unknown	[47]
miR-101	Upregulation			Unknown	[47]
miR-132	Upregulation			Unknown	[47]
miR-148	Downregulation			Unknown	[47]
miR-548	Upregulation			PB1, NS	[47]
miR-128	Upregulation	H5N1	A549	PA	[47]
miR-660	Upregulation		A549	PA	[47]
miR-16	Upregulation		A549	PB2, PB1, PA, NS1, NP, M	[47]
miR-181c	Upregulation		A549	BCL2, Il2, TNFα	[29]
miR-507	Unknown		Unknown	HA, PB2	[28]
miR-484	Downregulation		A549	furin	[53,54]
miR-24	Downregulation		A549	furin	[60]
miR-100	Downregulation	A/Thai/KAN1/2004	NCI-H292	IL13RA1, IL18RAP, CYTL1	[63]
miR-141	Upregulation	A/Thai/KAN1/2004	NCI-H292	CXCL12, TGFB2, CRLF3	[63]
miR-574-3p	Downregulation	A/Thai/KAN1/2004	NCI-H292	NDUFA42	[63]
miR-1274a	Downregulation	A/Thai/KAN1/2004	NCI-H292	TNFAIP8L2, TNFAIP3, BCL2L2, BCLAF1	[63]
miR-1274b	Downregulation	A/Thai/KAN1/2004	NCI-H292	TNFAIP8L2, IL1RAPL1, BCLAF1	[63]
miR-21	Downregulation	A/Thai/KAN1/2004	NCI-H292	CCl17, IL22, C20rf28, TNFSF13, CCL17, CCL19	[63]
miR-584-5p	Downregulation	A/Beijing/501/2009	A549	PB2	[46]
miR-1249	Downregulation	A/Beijing/501/2009	A549	PB2	[46]
miR-181c	Upregulation	A/Thai/KAN1/2004	NCI-H292	Unknown	[63]
miR-210	Upregulation	A/Thai/KAN1/2005	NCI-H292	Unknown	[63]
miR-29b	Upregulation	A/Thai/KAN1/2006	NCI-H292	Unknown	[63]
miR-483-3p	Upregulation	A/Thai/KAN1/2007	NCI-H292	Unknown	[63]
miR-324-5p	Upregulation	A/Thai/KAN1/2008	NCI-H292	Unknown	[63]
miR-663	Upregulation	A/Thai/KAN1/2009	NCI-H292	Unknown	[63]
miR-200a	Upregulation	A/Thai/KAN1/2010	NCI-H292	TGFβ	[63]
miR-1246	Upregulation	A/Thai/KAN1/2011	NCI-H292	Unknown	[63]
miR-146a	Downregulation	A/Vietnam/3212/04	Primary human macrophage	TRAF6	[64]
has-let-7	Differentially regulated based on time	A/Vietnam/3212/04	Primary human macrophage	IL8, MAPK11, MAPK8, MAP3K1	[64]
miR-485	Upregulation	A/duck/India/02CA10/2011 (H5N1)	HEK 293T, A549	PB1, RIG-1	[17]

### 2.2. miRNA Studies in NCI-H292 Cells, Human Macrophages and Human PBMCs

Human lung mucoepidermoid carcinoma cells, commonly known as NCI-H292 cells, are another cellular model commonly used to study IAV virus pathogenesis. MiR-141 is significantly upregulated by H5N1 IAV compared with H1N1 in infected NCI-H292 cells and has the capability to inhibit the expression of TGF-β2 (Figure 1). This was confirmed by showing that the inhibition of TGF-β2 could be reversed by using antagomiR-141. During the H5N1 IAV infection of NCl-H292 cells, miR-141, miR-181c, miR-210, miR29b, miR-483-3p miR-324-5p, and miR-663 showed significant upregulation compared with uninfected cells (Table 1). An increase in miR-141 was shown to repress TGF-β2 mRNA and protein expression (Figure 1) [63]. The 3′ untranslated region (UTR) of TGF-β2 mRNA harbors a binding site for miR-141/200a, and the introduction of miR-141/200a into cells leads to a notable decrease in TGF-β2 [65,66]. Another miRNA profiling study reported the upregulation of miR-5743P, miR663, and miR-1246 during H5N1 IAV infection [65]. Anti-inflammatory cytokines like TGF-β2 play a crucial role in regulating the inflammatory responses triggered by IAV infection, safeguarding the lung tissue from the harmful consequences of virus-induced inflammation. The reduction in TGF-β2 caused by miR-141 could represent an important step in the progression of excessive inflammation during H5N1 IAV infection (Figure 1). Another study using the NCI-H292 cell line found that H5N1 IAV infection altered the expression of miR-21, miR-100, miR-141, miR-574-3p, and miR-1274a and b (Table 1) [63].

MiRNA expression profiles in primary human monocyte-derived macrophages showed that 10 cellular genes are upregulated at the early stage of H5N1 IAV infection compared with H1N1 IAV infection due to their targeting miRNAs being downregulated [51]. The downregulation of the hsa-let-7 family of tumor suppressor miRNAs correlated with an increase in mRNA transcripts for genes in the RIG-I-like receptor signaling pathway. Another miRNA, miR-146a, is predicted to target TRAF6, a key signal transducer involved in RIG-I signaling. MiR-146a is significantly downregulated in response to H5N1 IAV infection compared with cells infected with H1N1 IAV (Table 1) [64]. It was also proposed that miR-146a is induced by toll-like receptor (TLR) 2, 4, and 5 ligands but not ligands that activate TLR-3, -7, and -9 [67]. A recent study showed that miR-146a expression is inversely correlated with TNF-α production [68]. MiR-146a is believed to play a crucial role in regulating pro-inflammatory signaling during the innate immune response [69]. Downregulation of miR-146a in response to H5N1 IAV infection could be a key factor driving the cytokine dysregulation observed in H5N1 influenza [70,71]. Studying this miRNA, particularly in vivo, may be crucial for understanding H5N1 IAV pathogenesis. This association highlights the regulatory functions of miRNAs in the cellular response to IAV infection, indicating that further research is needed to examine changes in miRNA expression levels that contribute to viral pathogenesis.

Ingle and colleagues [31] investigated the presence of target sites for miR-485 within the single-stranded RNA genome of H5N1 IAV. They identified a prominent target site located in the segment that encodes the RdRp catalytic subunit PB1 (Table 1). The miR-485 target site in H5N1 IAV is preserved across different HA subtypes collected from diverse regions and time periods. H5N1 IAV was found to induce the expression of miR-485 in a variety of primary cells and cell lines, including human small airway epithelial cells (SAECs) and human PBMCs, using microarray profiling and in silico screening. The dose-dependent bi-specificity of miR-485 for both RIG-I (Figure 1) and H5N1 PB1 revealed that endogenous miR-485 targets RIG-I mRNA in HEK 293T cells infected at a lower multiplicity of infection (MOI), which leads to the suppression of antiviral response (Table 1). However, in cells infected at a higher MOI, miRNA-485 changes its targets by binding with PB1, which causes a restriction in viral replication. Depending on the severity of the viral infection, the target of miR-485 shifts from RIG-I to PB1. This target switching suppresses the activation of innate antiviral responses during low-level influenza virus infections [31]. There is increasing evidence that viruses can decrease innate antiviral signaling through miRNA to evade the immune system [72,73].

## 3. In Silico Prediction-Based miRNA Studies

There are already plenty of software programs available for miRNA target prediction (e.g., TargetScan, Miranda), and each of them has unique parameters and algorithms. Three hundred differentially expressed miRNAs were individually analyzed in an in silico study for target sites in the complete sequence of the IAV (A/chicken/India/NIV33487/06(H5N1)) segment 8 encoding NS1. This analysis, which was performed using RNAhybrid 2.2, identified gga-miR-1658* as a miRNA that can potentially target the NS1 gene of the H5N1 IAV genome [74]. The prediction tool for host microRNAs that target viruses, called ViTA, uses the Miranda and TargetScan algorithms with a default minimum free energy (MFE) of −10 kcal/mol [75]. However, for the analysis, this study utilized a value of −24 kcal/mol because the lower the free energy, the firmer the binding structure is, and the more probable it is that the binding is genuine [74]. Another essential consideration during target prediction studies is that the specific target site must be accessible for the miRNA to function in the biological system, making accessibility criteria crucial. There might be targets, but if they are not accessible, they won’t have any effect on the system. For the secondary structure to be accessible at the target site where the miRNA binds, at least three consecutive bases must be unpaired, which increases the efficiency of prediction [76]. The NS1 protein, which is abundantly expressed in infected cells, functions to inhibit the host IFN response following IAV infection. As such, the NS1 gene is also referred to as an IFN antagonist [77], and it plays a crucial role in the establishment of H5N1 IAV infection. Therefore, targeting the NS1 gene can disrupt the pathogenesis of the avian AIV, making miRNA a promising tool for this intervention [77]. The NS1 protein of the H5N1 IAV may also be targeted by hsa-miR-138, hsa-miR-525-5p, and hsa-miR-124, which were identified as possible sequence-specific treatment agents. These miRNAs have been implicated in cancer and stress-related pathways, as well as the mTOR and MAPK pathways and host cell regulatory mechanisms [78].

The main site of H5N1 IAV replication in chickens is the lungs. A target prediction study identified miRNAs that may be able to bind to the transcript of H5N1 IAV segment 2 encoding PB1, PB1-F2, and N40. A set of 300 miRNAs expressed in chicken lungs were screened against the H5N1 HPAIV segment 2 using a variety of screening parameters. It is anticipated that chicken miRNAs gga-mir-133c, gga-mir-146c*, and gga-mir-1710 will target the expression of the N40, PB1-F2, and PB1 genes. These miRNAs can be leveraged in the development of strategies aimed at controlling avian influenza in chickens. Since the secondary structure of RNA is dynamic in living cells, it may be inferred that miRNAs exhibiting accessibility to the target site by both regional and global folding methods have a higher chance of effectively downregulating the target proteins. Further biological experiments, such as in vitro IAV challenge and luciferase reporter assays, are required to assess the biological relevance and effectiveness of these miRNAs for the prevention of H5N1 IAV replication in chickens [79].

Another study predicted that human miRNAs targeting several IAV subtypes, including H5N1, which may be helpful in blocking viral replication and improving knowledge of how IAV interacts with host cells. A total of 31 cellular miRNAs were examined for their ability to target H5N1 IAV (A/Thailand/NK165/2005) based on the outcome of hybridization pattern and pairing energy analysis between human miRNAs and their target viral gene. Furthermore, it was shown that the majority of these 31 miRNAs were directed toward the H5N1 IAV PB and PB2 genes, which included nine miRNAs targeting each. The only potential miRNA that could target the H1N1, H5N1, and H3N2 subtypes of the influenza A virus was hsa-miR-3145. Consequently, it is possible that hsa-miRNA3145 is a human cellular miRNA that broadly targets the IAV PB1 gene and is important in inhibiting viral replication [80].

According to a prediction-based study, H5N1 IAV may increase its pathogenesis by generating miRNAs that target genes implicated in important host cell processes. The generation of nine pre-miRNAs was predicted from the H5N1 viral genome after refining the sequences through computational miRNA prediction tools, iMiRNA-SSF and FOMmir (fixed-order Markov model based on the secondary structural pattern). Using the matureBayes and MiRduplexSVM algorithms, they also reported the location and sequencing of eighteen mature miRNAs within the validated precursor miRNA sequences. Viral miRNAs identified in this work may target genes that aid in the replication, transcription, and/or translation of the virus itself [81]. Several H5N1 IAV-encoded miRNAs were predicted by Li et al. (2018) and Hong et al. (2021), which could also advance our understanding of the host-virus interaction specifically with regard to exosomal miRNA expression against HPAIV H5N1 [82,83]. In summary, these studies highlight the potential of miRNAs, both host-derived and viral, as strategic tools for targeting key viral genes and disrupting the pathogenesis of H5N1 IAV, thus providing a promising avenue for developing novel antiviral therapies.

## 4. miRNA Studies in an In Vivo Chicken Model

A miRNA expression study performed on the lungs of broiler and layer chickens infected with an H5N3 IAV showed that gga-miR-146a was differentially expressed (upregulated) in the lungs of both infected broiler and layer chickens [84,85]. Three chicken miRNAs, gga-mir-1599, 1719, and 1594, were upregulated across three different detection methods: DESeq, Fisher’s exact test, and edgeR analysis. Seven miRNAs with differential expression patterns (including miR-34a, 30b, 202, 142-5p, 460b-5p, 449b, and 460a of gga-miR) were predicted to have binding sites for IL-17 receptor D in infected broiler chickens. Among these seven miRNAs, the first five were upregulated, and the last two were downregulated. The miRNA gga-miR-34a was exclusively expressed in the infected chicken lungs and was found to target the IAV genes HA, NA, PA, PB1, and PB2, as well as 14 immune-related genes. Some miRNAs that exhibit differential expression have numerous expected viral targets, suggesting a complex interaction between host miRNA expression and viral infection. For example, gga-miR-202 is predicted to target each of the eight AIV genes. A miRNA profile in layer chickens infected with H5N3 IAV found more downregulated miRNAs than upregulated miRNAs. In contrast, broiler chickens showed more upregulated miRNAs than downregulated miRNAs following IAV infection [84,85]. The major disparities between the two studies likely result from the genetic divergence between layer and broiler chickens, as these breeds have undergone extensive and varied selection over time with a focus on egg production and growth, respectively. Significantly elevated levels of miR-155 have been predicted to target the chicken anti-influenza gene MX1, potentially contributing to HPAIV infection in chickens. The c-Jun NH2-terminal kinases (JNK) pathway can be activated by miR-155 upregulation, which can induce apoptosis and may play a role in eliminating virus-infected cells [86,87]. To fully comprehend the mechanism of underlying miRNA regulation during IAV infection in chickens, further study is necessary.

The thymus and bursa of Fabricius are the key immunological organs in avian species, in charge of cell-mediated immunity and humoral immunity, respectively. The spleen also plays a crucial role in both innate and cell-mediated adaptive immunity during influenza virus infections [88]. Consequently, a study was conducted to investigate the miRNAs expressed in chicken and duck spleen, thymus, and bursa of Fabricius in the presence or absence of H5N1 HPAIV infection. Differences in miRNA repertoires and expression patterns in the immunological organs were observed between Specific Pathogen-Free (SPF) chickens and ducks [89]. In infected chickens, more miRNAs were upregulated in the immunological organs compared with ducks, where more miRNAs were downregulated [84,89]. Upregulated miRNAs in the chicken spleen, such as gga-miR-34c-5p, gga-miR-2188-5p, gga-miR-200b-5p, gga-miR-146b-5p and gga-miR-122-5p, targeted genes in the B Cell Receptor (BCR) signaling pathway. In the duck spleen, the downregulated miR-122-5p targets RASGRP3, which supports BCR signaling. The distinct disease presentations of chickens and ducks infected with H5N1 HPAIV may be greatly influenced by the divergent expression of miRNAs targeting immune genes [89].

Small RNA-seq analyses were carried out to characterize the expression of miRNAs in two different H5N1-infected Ri chicken lines (HPAIV-resistant and -susceptible) in order to determine the mechanisms by which IAV infection influences the expression of miRNAs linked to immune modulation [90]. In the susceptible line, gga-miR-146b-3p and gga-miR-27b-3p were elevated in infected trachea samples compared with control samples. In the resistant line, the IAV infection caused downregulation of gga-miR-451, which promoted the release of inflammatory cytokines and apoptosis [90]. The expression of proteins involved in the MAPK signaling cascade, including ZAK, MAP2K4, and TGFβ2, were suppressed by the upregulation of gga-miR-200a-3p in the resistant line [90,91]. Furthermore, in the resistant line, gga-miR-2188-5p (targeting PLK2) was downregulated, whereas gga-miR-200a-3p and gga-miR-203a (targeting MAP3K20 and MEF2c) were upregulated in the infected trachea samples. The results indicate that the immune response against IAV is significantly influenced by these differentially expressed miRNAs. These miRNAs modulate the IAV replication pathway, TGF-β, MAPK, and TLR signaling pathways, among other gene pathways implicated in IAV infection. Altogether, this research offers fresh perspectives on how miRNAs modulate the immune response against H5N1 HPAIV infection in chickens [90].

A total of miRNAs (miR-22-3p, miR-22-5p, miR-30e-5p, miR-31-5p, miR-32-5p, miR-33-5p, miR-92-5p, miR-155, miR-184-3p, miR-215-5p, miR-451, and let-7b) were shown to be differentially expressed in chicken lungs, immune organs, and embryo fibroblasts during infections with H5N3, H5N1, and H9N2 IAVs [84,89,92]. These differentially expressed miRNAs may play a crucial role in the interaction between chicken dendritic cells (DCs) and H9N2 IAV [82]. Studies have shown that miR-21-3p and miR-7 are upregulated in chicken DCs during H9N2 IAV infection. Two other miRNAs, miR-155 and miR-130b-3p, were reported to exhibit antiviral activity in chicken cells [93]. Further research is required to explore the precise function of these miRNAs during H9N2 infection in chicken DCs. In summary, the studies highlight the intricate roles of various miRNAs in modulating the immune response to highly pathogenic H5 strains of avian influenza viruses (AIV) in the in vivo models, emphasizing the complexity of host–pathogen interactions and the potential for miRNAs to serve as biomarkers or therapeutic targets in combating AIV infections.

## 5. Conclusions 

Investigation into the role of miRNAs in the response to HPIAV infection, particularly H5 subtypes, has yielded significant insights into the intricate interplay between host cellular mechanisms and viral pathogenesis. The findings from various studies underscore the pivotal role of miRNAs in modulating the immune response, regulating viral replication, and influencing viral pathogenesis in different models, including A549, NCI-H292, Vero cells, and in vivo chicken models.

Key miRNAs, including miR-203, miR-136, miR-188-3p, miR-21-3p, and miR-141, have been identified as crucial regulators of IAV infection. These miRNAs influence various pathways, such as IFN signaling, DNA demethylation, cytokine production, and viral RNA replication. For instance, miR-203 has been shown to suppress H5N1 IAV replication by targeting DR1, a host gene that facilitates viral RNA replication. Similarly, miR-136 acts as an endogenous activator of RIG-I, bolstering antiviral innate immunity. MiR-188-3p inhibits viral replication by targeting the PB2 gene of H5N6 and H7N9 IAVs. The differential expression of miRNAs in response to IAV infection has been highlighted in numerous independent studies, with significant variations observed between different subtypes of IAV (e.g., H5N1 vs. H1N1) and different cell types. These differences underline the complex regulatory networks at play and suggest potential targets for therapeutic intervention.

## 6. Recommendations for Future Research

1. Therapeutic development:
  - Targeted miRNA therapeutics: Given the demonstrated efficacy of certain miRNAs (e.g., miR-203, miR-136, miR-188-3p) in suppressing IAV replication, developing miRNA-based therapeutics could be a promising approach. These therapeutics could either mimic beneficial miRNAs or inhibit those that facilitate viral replication and pathogenesis.
  - Combination therapies: Combining miRNA therapeutics with existing antiviral drugs could enhance treatment efficacy and reduce the likelihood of resistance development.
2. Further research:
  - Mechanistic studies: More in-depth studies are needed to fully elucidate the mechanisms by which miRNAs regulate viral replication and host immune responses. Understanding these mechanisms will aid in the design of more effective therapeutic strategies.
  - In vivo validation: Conducting in vivo studies using animal models is crucial to validate the findings from in vitro studies. These studies will help in understanding the systemic effects of miRNA modulation and the potential side effects.
3. Diagnostic Tools:
  - miRNA profiling: Developing diagnostic tools that profile miRNA expression in response to IAV infection could aid in early detection and personalized treatment strategies. This could also help in monitoring the progression of the infection and the efficacy of therapeutic interventions.
4. Public health strategies:
  - Vaccination and monitoring: Continued emphasis on vaccination programs and monitoring of avian influenza outbreaks is essential. Integrating miRNA research with these programs could improve vaccine design and outbreak response strategies.

By focusing on these recommendations, researchers and healthcare professionals can harness the potential of miRNAs to develop innovative solutions for combating IAV infections, thereby improving public health outcomes and mitigating the impact of an influenza pandemic.

## Data Availability

Not applicable.
